# Impact of APOE4 dementia risk as a function of age in under‐represented groups using data from the *All of Us* Research program

**DOI:** 10.1002/alz.094631

**Published:** 2025-01-09

**Authors:** Valentina Ghisays, Ehsan Khajouei, Ignazio Piras, Dhruman D. Goradia, Michael H. Malek‐Ahmadi, Yinghua Chen, Marcus Naymik, Don Saner, Yi Su, Matthew J. Huentelman, Jason H Karnes, Eric M. Reiman

**Affiliations:** ^1^ Banner Alzheimer’s Institute, Phoenix, AZ USA; ^2^ College of Pharmacy, University of Arizona, Tucson, AZ USA; ^3^ The Translational Genomics Research Institute (TGen‐ an Affiliate of City of Hope), Phoenix, AZ USA

## Abstract

**Background:**

Recent studies have raised the possibility that APOE4 has a smaller impact on the risk for Alzheimer’s disease(AD) in non‐Hispanic Black(NHB) and Hispanic than non‐Hispanic White(NHW) individuals. Confirming that possibility in real‐world cohorts could have major implications for research and care in under‐represented groups(URGs). We used electronic health record (EHR) data from *All of Us (*AoU) to compare prevalence of all‐cause dementia in these URGs and the impact of APOE4 carriage and allelic dose on dementia hazard ratios.

**Methods:**

We used EHR data from **10,045** Hispanic‐White, **14,937** NHB, and **60,388** NHW ages 60+ to characterize the risk of converting to a diagnosis of dementia in APOE4 homozygotes(HM), heterozygotes(HT), combined carriers, and non‐carriers(NC) using Cox hazards‐ratio(HR) and 95%CIs. We used genomic data to extract genotypes and all ICD‐9 codes related to dementia diagnosis or associated drug prescriptions to identify cases and controls.

**Results:**

Results showed that APOE4 increased dementia risk significantly in all groups but varied in race and ethnicity by allelic frequency. In NHB individuals, APOE4 carriers had a significant increased risk (HR 1.25, 95%CI 1.00‐1.57, p = 0.04), while we failed to detect differences in HM and HTs. Hispanic carriers and HTs also exhibited significant risk (HR 1.54, 95%CI 1.24‐1.90; HR 1.52, 95%CI 1.22‐1.88, p<0.0001; respectively) but failed to detect differences in HMs. In NHW, risk increased significantly with APOE4 copy (HT: HR 1.42, 95%CI 1.28‐1.58; HM: HR 3.70, 95%CI 2.92‐4.68; combined carriers: HR 1.55, 95%CI 1.40‐1.72, p<0.0001). Compared to NHW, Hispanic and NHB groups had 2.4x and 1.6x higher risk (HR 2.44, 95%CI 2.19‐2.72; HR 1.55, 95%CI 1.38‐1.75, p<0.0001).

**Conclusion:**

Using diagnostic codes in a large number of AoU participants, we observed greater risk of dementia in URGs and trends to support APOE4 allelic dose effects overall and greater effects in NHWs. Limitations in detecting APOE4 allelic dose effects or interactions with these URGs could be related to sample size, accuracy to characterize AD cases and controls in EHR datasets, and any inaccuracies/biases in clinical diagnosis. This study provides support to further characterize prevalence of AD and non‐AD dementias and impact of APOE4 on AD dementia risk using plasma pTau217 as an AD endophenotype.

